# Estimating ^131^I biokinetics and radiation doses to the red marrow and whole body in thyroid cancer patients: probe detection versus image quantification*

**DOI:** 10.1590/0100-3984.2015.0079

**Published:** 2016

**Authors:** José Willegaignon, Rogério Alexandre Pelissoni, Beatriz Christine de Godoy Diniz Lima, Marcelo Tatit Sapienza, George Barberio Coura-Filho, Marcelo Araújo Queiroz, Carlos Alberto Buchpiguel

**Affiliations:** 1 PhD, Chief Medical Physicist, Instituto do Câncer do Estado de São Paulo Octavio Frias de Oliveira (Icesp), São Paulo, SP, Brazil.; 2 Technologist, Instituto do Câncer do Estado de São Paulo Octavio Frias de Oliveira (Icesp), São Paulo, SP, Brazil.; 3 PhD, Assistant Professor, Radiology Department, Faculdade de Medicina da Universidade de São Paulo (FMUSP), São Paulo, SP, Brazil.; 4 MD, Nuclear Physician, Instituto do Câncer do Estado de São Paulo Octavio Frias de Oliveira (Icesp), São Paulo, SP, Brazil.; 5 MD, Radiologist Physician, Instituto do Câncer do Estado de São Paulo Octavio Frias de Oliveira (Icesp), São Paulo, SP, Brazil.; 6 PhD, Full Professor, Radiology Department, Faculdade de Medicina da Universidade de São Paulo (FMUSP), São Paulo, SP, Brazil.

**Keywords:** Thyroid neoplasms, Iodine radioisotopes/therapeutic use, Radioisotopes/pharmacokinetics, Dosimetry, Radiotherapy

## Abstract

**Objective:**

To compare the probe detection method with the image quantification method when estimating ^131^I biokinetics and radiation doses to the red marrow and whole body in the treatment of thyroid cancer patients.

**Materials and Methods:**

Fourteen patients with metastatic thyroid cancer, without metastatic bone involvement, were submitted to therapy planning in order to tailor the therapeutic amount of ^131^I to each individual. Whole-body scans and probe measurements were performed at 4, 24, 48, 72, and 96 h after ^131^I administration in order to estimate the effective half-life (T_eff_) and residence time of ^131^I in the body.

**Results:**

The mean values for T_eff_ and residence time, respectively, were 19 ± 9 h and 28 ± 12 h for probe detection, compared with 20 ± 13 h and 29 ± 18 h for image quantification. The average dose to the red marrow and whole body, respectively, was 0.061 ± 0.041 mGy/MBq and 0.073 ± 0.040 mGy/MBq for probe detection, compared with 0.066 ± 0.055 mGy/MBq and 0.078 ± 0.056 mGy/MBq for image quantification. Statistical analysis proved that there were no significant differences between the two methods for estimating the T_eff_ (*p* = 0.801), residence time (*p* = 0.801), dose to the red marrow (*p* = 0.708), and dose to the whole body (*p* = 0.811), even when we considered an optimized approach for calculating doses only at 4 h and 96 h after ^131^I administration (*p* > 0.914).

**Conclusion:**

There is full agreement as to the feasibility of using probe detection and image quantification when estimating ^131^I biokinetics and red-marrow/whole-body doses. However, because the probe detection method is inefficacious in identifying tumor sites and critical organs during radionuclide therapy and therefore liable to skew adjustment of the amount of ^131^I to be administered to patients under such therapy, it should be used with caution.

## Abstract (pt)

**Objetivo:**

Comparar o desempenho dos métodos de detecção de sonda e quantificação de imagens na estimativa da biocinética do radioisótopo ^131^I e das doses de radiação na medula óssea vermelha e no corpo inteiro durante a radioiodoterapia em pacientes com câncer de tireoide.

**Materiais e Métodos:**

Catorze pacientes portadores de câncer metastático de tireoide, sem acometimento ósseo, foram submetidos ao planejamento terapêutico visando estabelecer a melhor atividade de ^131^I a ser empregada na radioiodoterapia. Imagens cintilográficas e captações de corpo inteiro foram adquiridas 4, 24, 48, 72 e 96 h após a administração de atividades traçadoras de ^131^I, visando estimar a meia-vida efetiva (T_1/2ef_) e o tempo de residência do ^131^I no organismo dos pacientes.

**Resultados:**

Os valores médios de T_1/2ef_ e tempo de residência foram, respectivamente, 19 ± 9 h e 28 ± 12 h pelo método de detecção de sonda e 20 ± 13 h e 29 ± 18 h pela quantificação de imagens. As doses médias na medula óssea vermelha e no corpo inteiro foram, respectivamente, 0,061 ± 0,041 mGy/MBq e 0,073 ± 0,040 mGy/MBq pelo método de detecção de sonda e 0,066 ± 0,055 mGy/MBq e 0,078 ± 0,056 mGy/MBq pela quantificação de imagens. A análise estatística demonstrou que os dois métodos apresentam desempenho semelhante no tocante à estimativa de T_1/2ef_ (*p* = 0,801), tempo de residência (*p* = 0,801) e doses, tanto na medula óssea vermelha (*p* = 0,708) como no corpo inteiro (*p* = 0,811), mesmo com métodos otimizados de dosimetria que levam em consideração somente dois pontos de medida (4 h e 96 h) após a administração de ^131^I (*p* > 0,914).

**Conclusão:**

Existe excelente concordância entre o método de detecção de sonda e a quantificação de imagens quanto à estimativa da biocinética do ^131^I e das doses absorvidas de radiação. Contudo, o método de detecção de sonda deve ser usado com cuidado por ser incapaz de identificar regiões metastáticas e órgãos críticos durante a terapia com radionuclídeos, podendo distorcer ajustes da atividade de ^131^I a ser administrada durante a radioiodoterapia.

## INTRODUCTION

The management of patients with differentiated thyroid cancer involves radioiodine therapy to ablate remnants of thyroid tissues after surgical resection of the gland or in the treatment of metastases^(1)^. A large amount of ^131^I is generally administered to patients during the treatment of metastatic diseases. Under these circumstances, it would be more appropriate to tailor the amount according to individual needs and to the radiotoxic effect on healthy organs, such as the red marrow, lungs, kidneys, and salivary glands^(2,3)^.

Several of the dosimetric methods used for adjusting the amount of ^131^I to be administered in therapy are based on delivering a maximum radiation dose of 2-3 Gy to red-marrow tissues, while abiding by the rules for radioiodine-avid lung metastases^(2,4)^. When determining the radiation dose per unit of ^131^I activity (mGy/MBq) to be received by the red marrow and whole body, sequential measurements of the circulating levels of ^131^I are generally required. Typically, the circulating level of ^131^I is either estimated through invasive procedures, such as the collection and analysis of blood samples, or inferred from whole-body radiation measurements with a radiation detection probe or by image quantification^(5,6)^.

Considering the radiation detection probe and image quantification methods as two different procedures, to be used as alternatives for determining radiometric data, our aim here was to compare their performance when estimating ^131^I biokinetics and radiation doses to the whole body and to the red marrow.

## MATERIALS AND METHODS

### Patient characteristics and radiometric data acquisition

Fourteen patients with metastatic differentiated thyroid cancer were submitted to a dosimetric protocol in order to tailor the amount of ^131^I to be administered in individual therapy. None of the patients showed evidence of distant metastasis. The study was approved by the local research ethics committee.

Prior to ^131^I administration, patients underwent 4-6 weeks of thyroid hormone withdrawal and were maintained on an iodine-poor diet, in order to raise the endogenous thyroid-stimulating hormone level (to > 30 mIU/L) and stimulate ^131^I uptake by thyroid tissue remnants and metastases. In the present study, exogenous recombinant human thyroid-stimulating hormone was not administered.

The proportional whole-body ^131^I retention was calculated at 4, 24, 48, 72, and 96 h after ^131^I administration with a radiation detection probe and a gamma camera. Patient data and measurement times were documented.

### Radiation detection probe measurements

**Figure 1:**
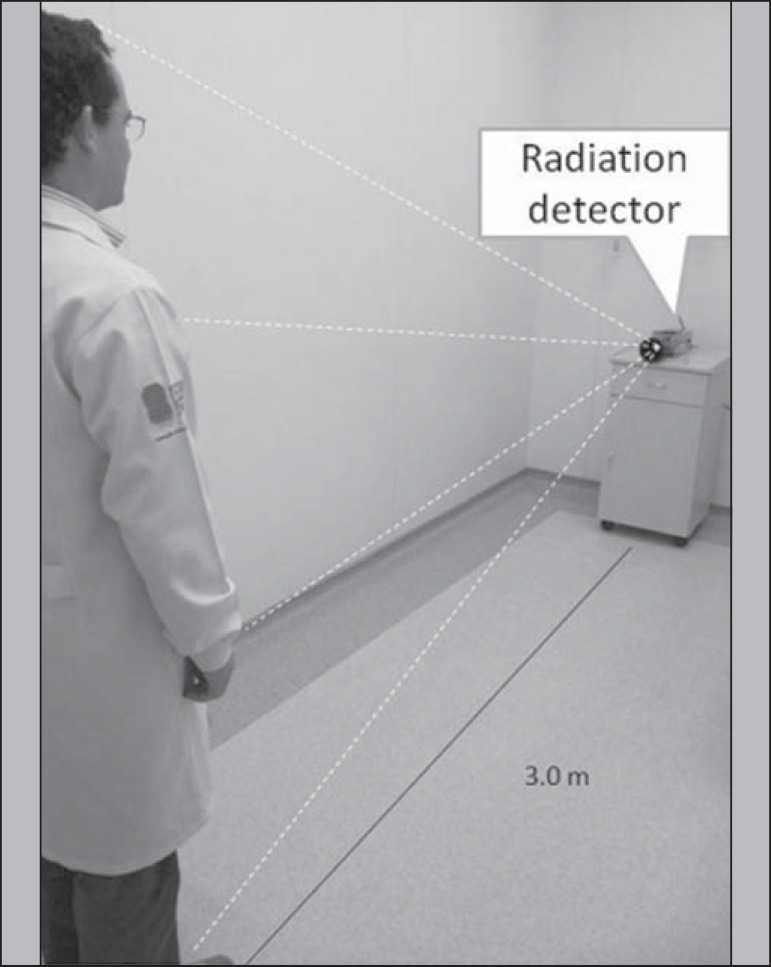
Schematic arrangement for measuring radiometric data acquired with the probe detection method.

An NaI radiation detector (identiFINDER^TM^ Digital Spectrometer; Thermo Fisher Scientific Inc., Erlangen, Germany), with a 35 mm × 51 mm crystal, was used. The individuals were placed in a standing position 3.0 m from the detector, as illustrated in Figure 1. Each measurement was taken three times, for 3 min each time, in the same location (an area with a low background radiation level) every day. The net radioactivity (counts/min) was determined only for the anterior acquisition. Before each patient measurement, the background radiation was measured (also for 3 min) in the absence of the patient. Although partial shielding of the detector was considered propitious, the shielding between the patient and the NaI crystal was removed in order to reduce background radiation. The solid angle of the detector was sufficient to receive photons from the entire body of the patient. The duration of the measurement was sufficient to providing net counts > 10^5^ at each measuring point, even when the background radiation was subtracted.

### Image acquisition

**Figure 2:**
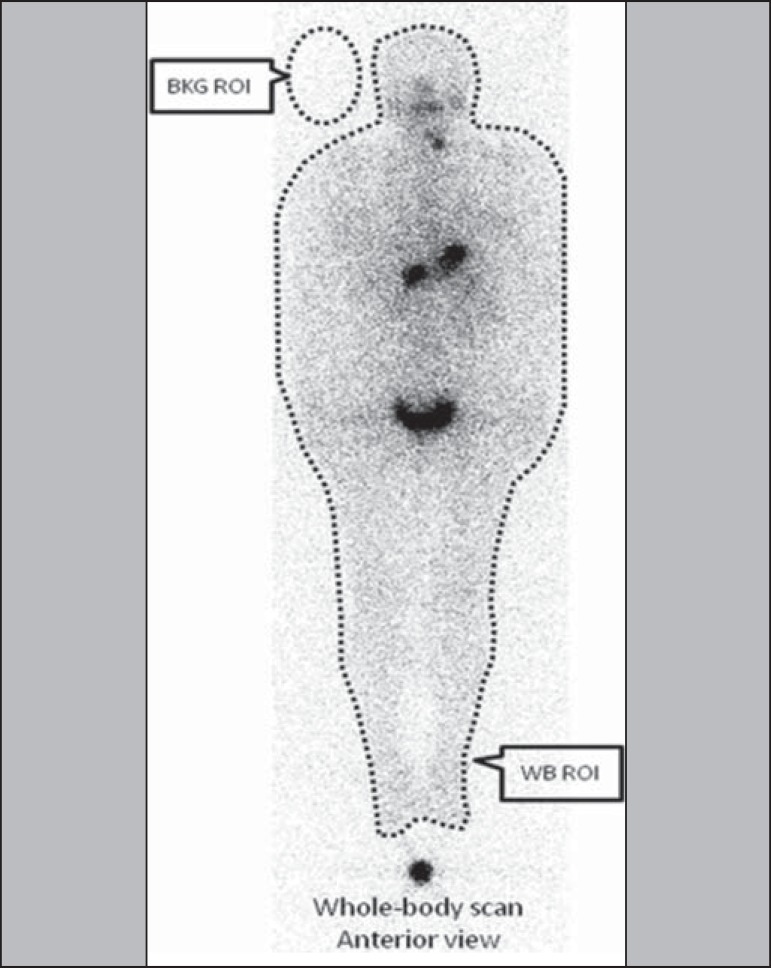
Schematic arrangement for measuring radiometric data acquired with the image quantification method

A dual-head gamma camera-for single-photon emission computed tomography/computed tomography (SPECT/CT)-with a high-energy collimator was used in order to estimate the ^131^I activity within the body, by whole-body image quantification and as a function of time. Only planar nuclear medicine images were used for quantification. As depicted in Figure 2, the activity was estimated on the basis of the radiation counts for designated regions of interest (ROIs) around the whole body, minus the background radiation, as follows:

$$\mathrm{WBnet}=\mathrm{WBpixels}\times \left(\mathrm{WBcts}/\mathrm{pixel}-\mathrm{BKGcts}/\mathrm{pixel}\right)$$

where *WBnet* is the net whole-body count, *WBpixels* is the total number of pixels for the whole-body ROI in question, *WBcts/pixel* is the average count per pixel for the whole-body ROI in question, and *BKGcts/pixel* is the average count per pixel of background radiation.

A copy of the ROIs (WB and BKG) from the first images was replaced in later images. The same calculation method was applied to each of the five images acquired from each patient. The SPECT/CT was provided with the software Syngo MI Applications 2009A^®^ (Siemens Medical Solutions USA, Inc.; Malvern, PA, USA). Whole-body planar images were acquired in a 256 × 1024 matrix with a scan speed of 8 cm/min. All acquired images were analyzed using the free software Image J, version 1.45s (Wayne Rasband, National Institutes of Health; Bethesda, MD, USA).

One patient was used as a standard source for evaluating the remaining whole-body activity as a function of time after ^131^I administration, net whole-body counts being normalized to the first data point (taken as 100%). After ^131^I administration, the patients were allowed to void only after the first probe measurement and image acquisition. When estimating ^131^I biokinetics and radiation doses, we considered only the anterior view (for probe measurement and image acquisition), given that our experience has shown that there is little difference between the data estimated with this methodology and those estimated by considering conjugate views (anterior and posterior acquisition). Based on whole-body scanning, the net count rate from anterior acquisition only is, on average, approximately 5% superior to that of conjugate acquisition, indicating that determining only the anterior count rate is a practical and easy method for evaluating radioiodine retention as a function of elapsed time after ^131^I administration.

### Red-marrow and whole-body absorbed dose calculation

For each patient, the cumulative whole-body activity (*Ã_wb_*) was calculated by applying the following equation:

$${Ã}_{\mathrm{wb}}=\left(1.443\times A_{0}\times T_{\mathrm{eff}}\right)$$

where *A_0_* is the amount of radioactivity administered, and *T_eff_* is the effective whole-body half-life of ^131^I.

To describe the radiometric data plotted on the graph "estimated whole-body activity *versus* time of measurement", the *T_eff_* was determined by a simple exponential function adjustment:

$$y=a+{\mathrm{be}}^{-\lambda_{\mathrm{eff}}t}$$

where *λ_eff_* = 0.693 / *T_eff_*.

According to the radiometric data acquired by probe detection or image quantification, *Ã_wb_, T_eff_*, and residence time were calculated in duplicate for each patient. The residence time was estimated by dividing the *Ã_wb_* by the total amount of ^131^I administered (*A_0_*).

The absorbed dose of radiation per unit of ^131^I activity (mGy/MBq), estimated for the whole body and the red marrow, was calculated with OLINDA/EXM computer soft-ware^(7)^. We estimated the absorbed dose by introducing the ^131^I residence time into the computer program, choosing a specific adult anthropomorphic phantom (adult male or adult female) ^131^I radionuclide, and adjusting the parameters of the software according to patient body mass and thyroid tissue mass. The last was estimated by calculating the total ^131^I uptake by remnant tissue after thyroidectomy and considering 1% of the ^131^I uptake per gram of tissue. As described in a previous study^(8)^, the red-marrow dose was adjusted according to patient weight.

## RESULTS

Of the 14 patients evaluated, two were male and 12 were female. Patient ages ranged from 25 to 63 years. All of the patients had previously undergone total or near-total thyroidectomy. The mean value ± 1 standard deviation for patient weight was 77 ± 19 kg, and the mean height was 1.63 ± 0.06 m. The activity of the tracer ^131^I ranged from 74 MBq to 126 MBq, with a mean value of 87 ± 14 MBq, administered to patients (orally in liquid form) according to a dosimetric protocol and as an aid in staging the disease. Patient characteristics are presented in Table 1.

**Table 1:** Patient characteristics and units of ^131^I activity administered to patients for dosimetric purposes.

		Age	Weight	Height	^131^I activity
Patient	Gender	(years)	(kg)	(m)	(MBq)
P1	Female	63	66.6	1.62	74.00
P2	Female	31	124.0	1.58	82.51
P3	Male	59	65.0	1.69	93.24
P4	Female	49	72.1	1.66	86.95
P5	Female	53	103.0	1.57	126.17
P6	Female	27	81.8	1.66	85.10
P7	Female	37	73.4	1.55	85.84
P8	Female	50	76.5	1.56	74.74
P9	Female	25	43.0	1.69	78.07
P10	Female	51	67.5	1.66	74.37
P11	Female	45	72.9	1.61	77.33
P12	Male	47	67.6	1.58	103.23
P13	Female	26	77.2	1.72	86.95
P14	Female	32	88.9	1.69	92.50
Mean value		42.50 ±	77.02 ±	1.63 ±	87.21 ±
		12.68	19.04	0.06	13.99

**Figure 3:**
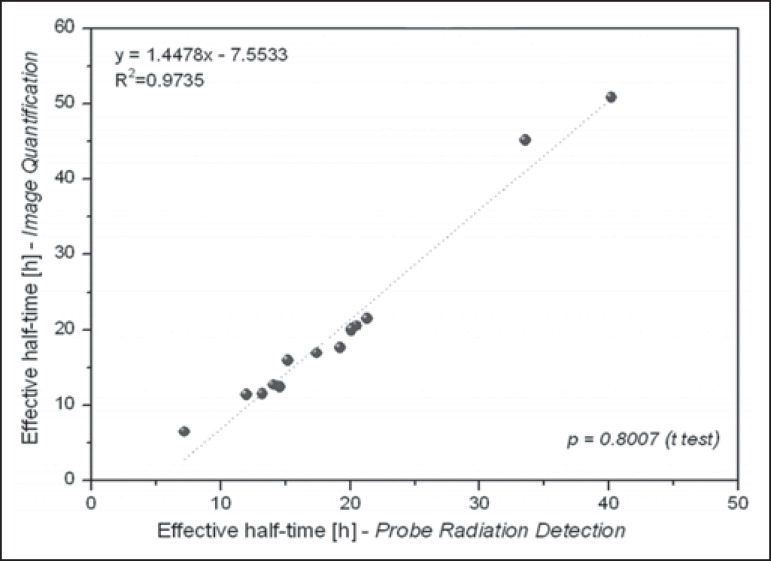
Correlations between effective half-lives calculated from radiometric data acquired with the probe detection and image quantification methods.

**Figure 4:**
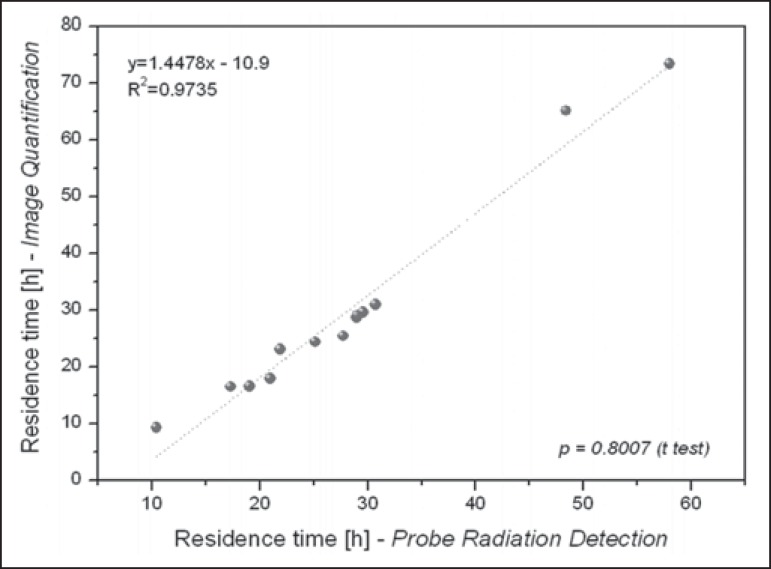
Correlations between residence times calculated from radiometric data acquired with the probe detection and image quantification methods.

Table 2 presents the effective half-life and residence time of ^131^I for each patient, determined by considering radiometric data acquired by means of probe detection and image quantification. The mean half-life and residence time values were 19 ± 9 h and 28 ± 12 h, respectively, for probe detection, compared with 20 ± 13 h and 29 ± 18 h, respectively, for image quantification. The effective half-life calculated from image quantification was, on average, 9.38% higher than that calculated from probe measurements, the difference ranging from 0.09% to 34.53% among the patients. A similar difference was observed in residence times. As can be seen in Figures 3 and 4, no significant difference was found between the probe detection and image quantification methods in terms of the calculated effective half-lives or residence times (*p* = 0.801 for both).

**Table 2:** Estimated effective half-life and residence time of ^131^I when considering individually based radiometric data acquired with probe detection and planar image quantification.

	Probe detection			Image quantification	
	Effective	Residence		Effective	Residence
Patient	half-life (h)	time (h)		half-life (h)	time (h)
P1	20.50	29.58		20.53	29.62
P2	15.18	21.90		15.99	23.07
P3	40.20	58.01		50.84	73.36
P4	19.22	27.73		17.62	25.43
P5	21.30	30.73		21.49	31.01
P6	14.54	20.98		12.41	17.91
P7	13.20	19.05		11.50	16.59
P8	20.09	28.99		19.90	28.72
P9	20.12	29.03		20.14	29.06
P10	7.20	10.39		6.48	9.35
P11	17.43	25.15		16.90	24.39
P12	33.54	48.40		45.12	65.11
P13	11.98	17.29		11.44	16.51
P14	14.05	20.27		12.71	18.34
Mean value	19.18 ±	27.68 ±		20.22 ±	29.18 ±
	8.57	12.36		12.57	18.14

**Figure 5:**
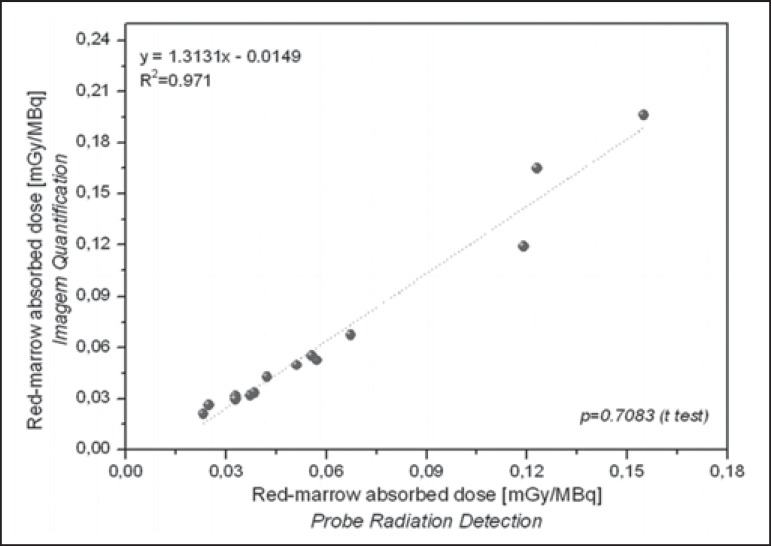
Correlations between radiation doses to the red marrow, as calculated from radiometric data acquired with the probe detection and image quantification methods.

**Figure 6:**
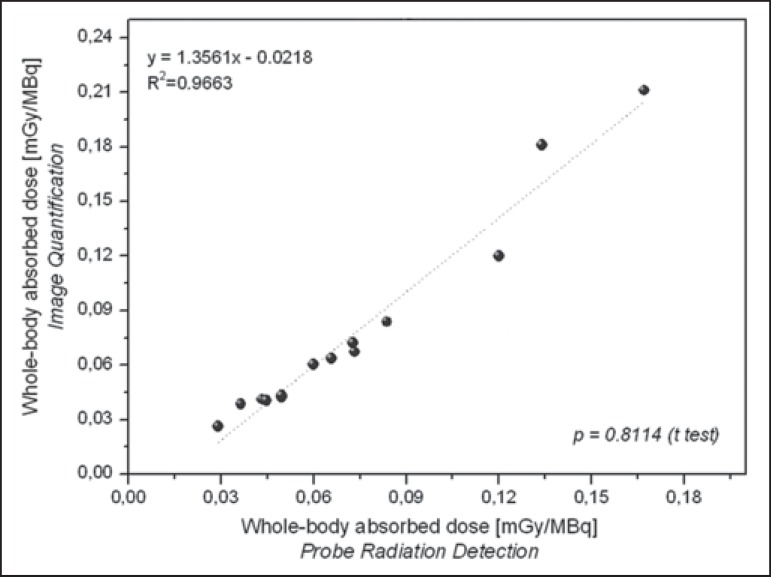
Correlations between radiation doses to the whole body, as calculated from radiometric data acquired with the probe detection and image quantification methods.

Radiation doses to the red marrow and whole body per unit of ^131^I activity administered are presented in Table 3. Mutual correlations, when estimated by probe detection or image quantification, are shown in Figures 5 and 6. When estimated by probe detection, the mean red-marrow and whole-body doses were 0.061 ± 0.041 mGy/MBq and 0.073 ± 0.040 mGy/MBq, respectively, compared with 0.066 ± 0.055 mGy/MBq and 0.078 ± 0.056 mGy/MBq, respectively, when estimated by image quantification. The mean differences between the probe detection and image quantification methods in terms of the radiation doses calculated was similar to those found for the effective half-lives and residence times. The statistical analysis revealed no significant difference between the two methods, in terms of the estimated radiation doses to the red marrow (*p* = 0.708) and to the whole body (*p* = 0.811).

**Table 3:** Estimation of red-marrow and whole-body radiation doses when considering radiometric data from probe detection and planar image quantification.

	Probe detection			Image quantification	
	Red-marrow	Whole-body		Red-marrow	Whole-body
Patient	(mGy/MBq)	(mGy/MBq)		(mGy/MBq)	(mGy/MBq)
P1	0.067	0.084		0.067	0.084
P2	0.025	0.036		0.026	0.038
P3	0.155	0.167		0.196	0.211
P4	0.057	0.073		0.052	0.067
P5	0.042	0.060		0.043	0.060
P6	0.037	0.050		0.032	0.042
P7	0.038	0.050		0.033	0.043
P8	0.056	0.073		0.055	0.072
P9	0.119	0.120		0.119	0.120
P10	0.023	0.029		0.021	0.026
P11	0.051	0.066		0.050	0.064
P12	0.123	0.134		0.165	0.181
P13	0.033	0.043		0.031	0.041
P14	0.033	0.045		0.030	0.040
Mean value	0.061 ±	0.073 ±		0.066 ±	0.078 ±
	0.041	0.040		0.055	0.056

**Figure 7:**
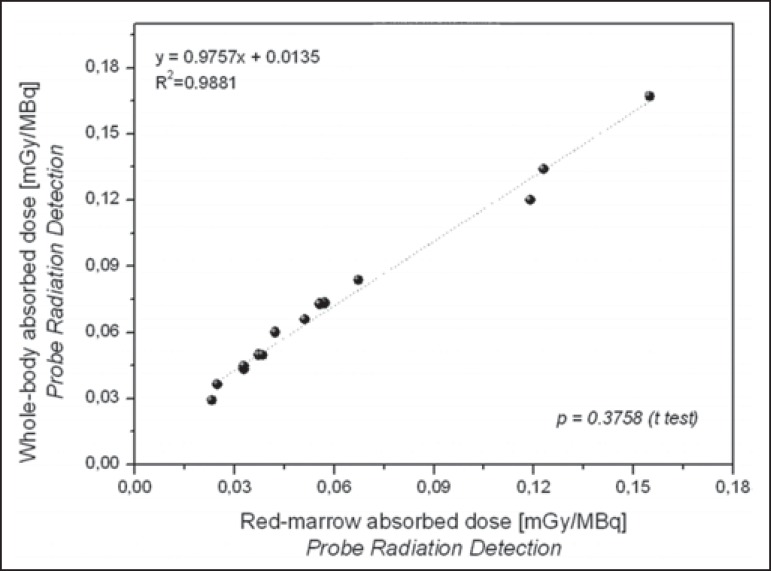
Correlations between radiation doses to the red marrow and radiation doses to the whole body when only radiometric data acquired with probe detection were considered.

**Figure 8:**
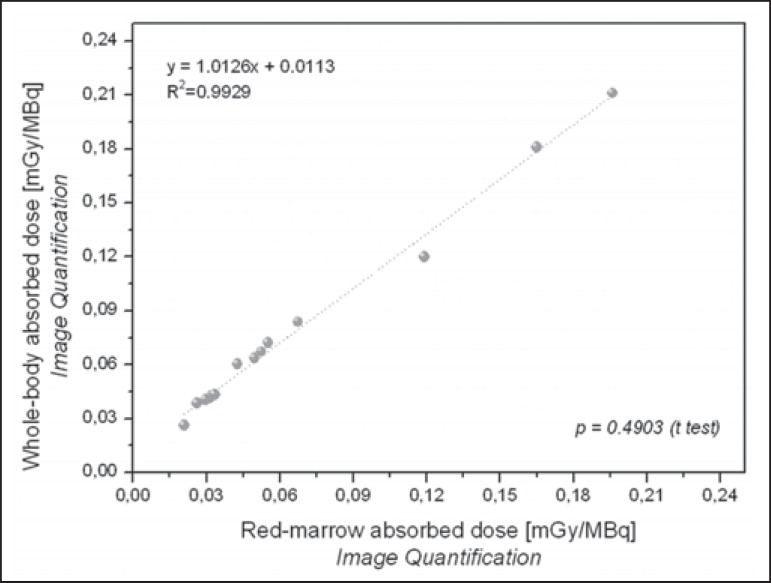
Correlations between radiation doses to the red marrow and radiation doses to the whole body when only radiometric data acquired with image quantification were considered.

As can be seen in Figures 7 and 8, there were a good correlation between the estimated doses delivered to the red marrow and whole body for the same patient, as calculated by probe detection (*p* = 0.376; *R^2^* = 0.988) and image quantification (*p* = 0.490; *R^2^* = 0.993). Overall (as calculated by both methods), the mean radiation dose per unit of ^131^I activity administered received by the whole body was approximately 27% higher than that received by the red marrow (range, 0.84-44.0%).

**Figure 9:**
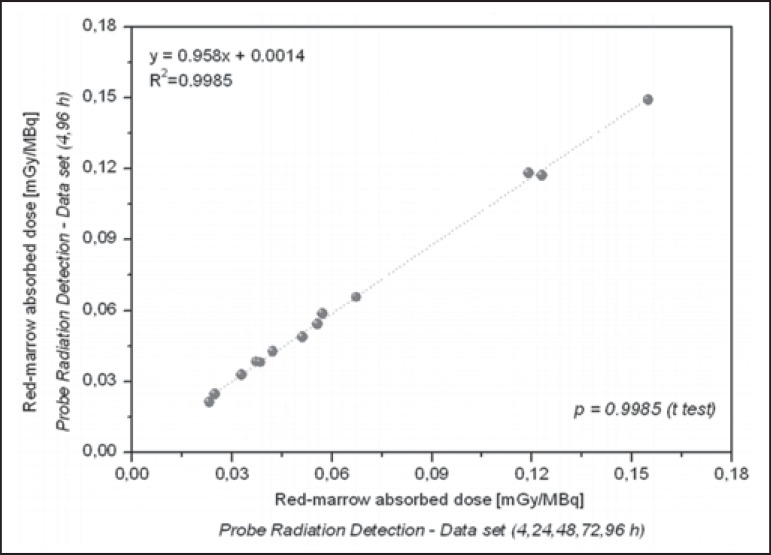
Correlations between radiation doses to the red marrow calculated by considering measurements obtained at five time points (4, 24, 48, 72, and 96 h) and those calculated by considering measurements obtained at only two (4 h and 96 h), using only radiometric data acquired with the probe detection method.

**Figure 10:**
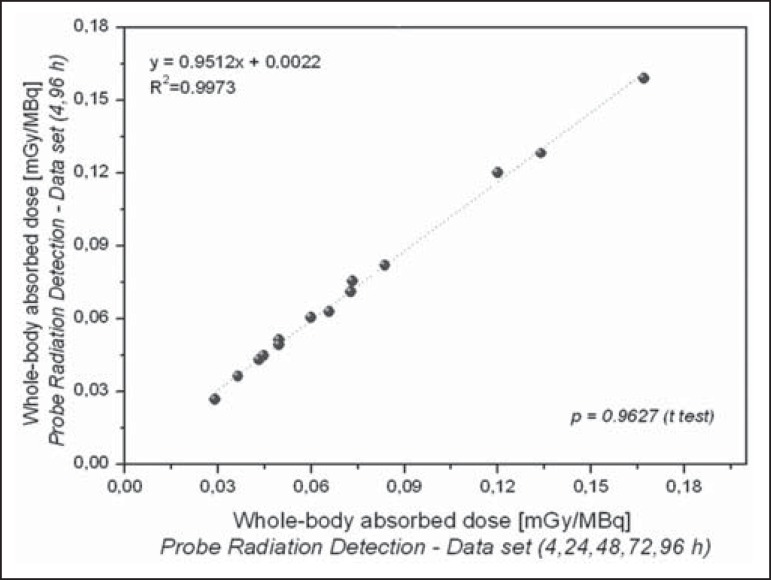
Correlations between radiation doses to the whole body calculated by considering measurements obtained at five time points (4, 24, 48, 72, and 96 h) and those calculated by considering measurements obtained at only two (4 h and 96 h), using only radiometric data acquired with the probe detection method.

**Figure 11:**
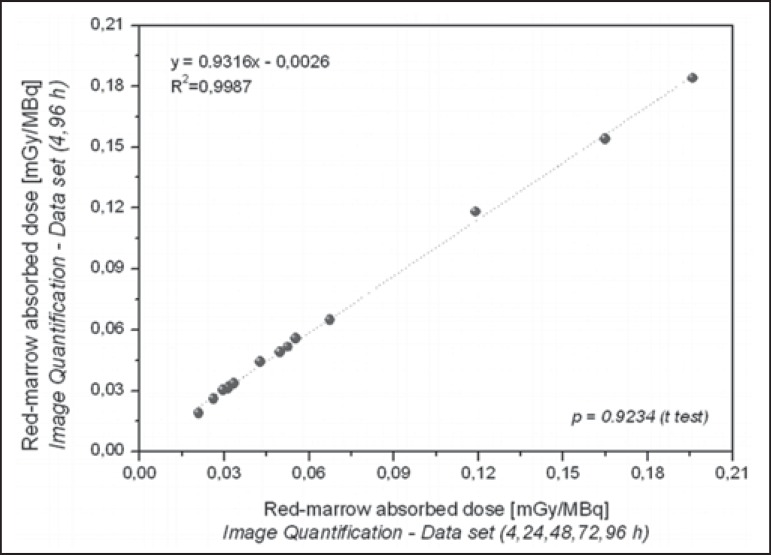
Correlations between radiation doses to the red marrow calculated by considering measurements obtained at five time points (4, 24, 48, 72, and 96 h) and those calculated by considering measurements obtained at only two (4h and 96 h), using only radiometric data acquired with the image quantification method.

**Figure 12:**
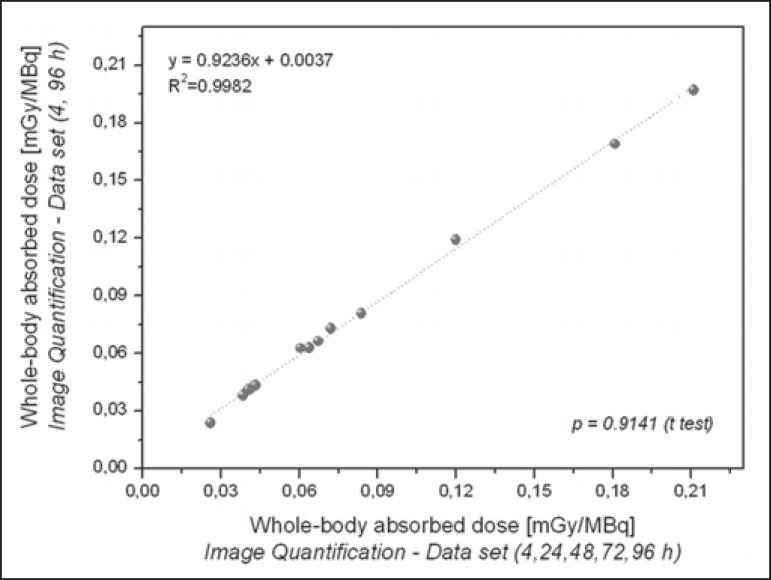
Correlations between radiation doses to the whole body calculated by considering measurements obtained at five time points (4, 24, 48, 72, and 96 h) and those calculated by considering measurements obtained at only two (4 h and 96 h), using only radiometric data acquired with the image quantification method.

The radiation doses presented in Table 3 were calculated by considering measurements obtained at five time points (4, 24, 48, 72, and 96 h). However, an optimized approach can be applied, given that similar dose estimation results are obtainable when using only the measurements obtained at the two extremes (4 h and 96 h). Correlations between the optimized and the non-optimized method can be seen in Figures 9, 10, 11 and 12. Statistical analysis with a t-test showed that there was no significant difference between the two methods for estimating doses (*p* > 0.914).

It is important to note that, when estimating the radiation doses to the red marrow and whole body in this study, we did not take into consideration doses received by the patients less than 4 h after ^131^I administration. Soon after oral administration, ^131^I is mainly accumulated in the stomach, and requires a certain time before entering into blood circulation. It is assumed that immediate whole-body ^131^I dispersion would lead to incorrect radiation dose estimation. However, if one considers immediate ^131^I dispersion in the first 4 h, the dose to the red marrow or whole body would account for only approximately 10% of the total dose received by the two. However, this assumption was not made in the present study, because the main aim here was to compare the performance of probe detection and image quantification in radiation dose estimation.

## DISCUSSION

Since 1962, when Benua et al.^(9)^ presented a study on radioiodine dosimetry for metastatic thyroid cancer patients, many papers on the same topic have been published^(8)^. However, there is still controversy regarding this point, due to a scarcity of studies comparing dose-response correlations according to the dosimetric method employed. In most dosimetric methods, consideration (for dose calculation) is given only to the use of extensive radiometric data from patients undergoing diagnostic or therapeutic procedures. Nevertheless, these data can be acquired by collecting and measuring radiometric data from body fluids or even inferred from body radiation measurement.

According to the European Association of Nuclear Medicine Dosimetry Committee blood and bone marrow dosimetry guidelines for differentiated thyroid cancer^(5)^, the probe and image quantification methods are considered as alternative procedures for estimating the amounts of radioactive iodine inside the body and provide similar absorbed-dose results when those data are used for internal dose calculation. However, from our point of view, there have been no studies presenting dosimetric data in a satisfactory way to confirm this assumption. In addition, the limitations of using only radiometric data acquired by probe detection to tailor the ^131^I amount to be administered to metastatic thyroid cancer patients have not been well addressed. In this context, the present study makes a contribution to internal dosimetry by furnishing comparisons of dosimetric data estimated by probe detection and image quantification.

On the basis of the results of the current study, and by applying the methodology described here, we can state that the probe detection and image quantification methods provided similar results when estimating ^131^I effective half-life and residence time within the body. Therefore, both methods can be considered valid for determining these parameters. The differences between the two methods when estimating radiation doses per unit of ^131^I activity administered, either to the red marrow or to the whole body were not statistically significant. The mean radiation dose to per unit of ^131^I activity administered was considerably higher for the whole-body doses than for the red-marrow doses, and the difference was similar to that reported in other studies^(8)^. It is important to note that, depending on the physical characteristics of each patient (e.g., weight and height), that difference could reach values even higher than the 27% observed in the present study, which is a representative value for a standard patient (70 kg in weight and 1.70 m tall), hence the impropriety of using that value for every patient without some kind of adjustment according to patient biotype, especially in the case of patients presenting bone metastases.

When determining appropriate doses, good correlations were found between the absorbed doses determined at five time points (4, 24, 48, 72, and 96 h) and those determined at only two (4 h and 96 h), when either probe detection or image quantification was used. Reducing the number of data points necessary for estimating radiation doses implies a general reduction in the costs involved in nuclear medicine therapy planning, while working in favor of implementing this procedure in the daily routine of a nuclear medicine department. A similar study of optimizing the amount of radiometric data necessary for dosimetry in Graves' disease therapy was previously conducted by our group using the probe detection method^(10,11)^. Another group of authors used positron emission tomography images to estimate the radiation doses given to patients with thyroid cancer^(12)^. Both studies were in agreement, in that it is possible to achieve a considerable reduction in the amount of radiometric data required for providing adequate dosimetry. However, it is possible that more data are needed in the case of tumor dosimetry.

The probe detection and image quantification methods provide similar results in determining radiometric data for planning red-marrow and whole-body dosimetry for thyroid cancer patients without metastatic bone involvement. However, the probe detection method should be used with a certain degree of caution, given that, in some clinical cases, the red-marrow is not the first organ at risk during therapy, especially in patients who present with regional or disseminated diseases. Unlike image quantification, probe detection is unable to identify tumor sites or evaluate the ^131^I biokinetics within specific organs, such as the kidneys, lungs, and brain, or in tumors surrounding the spinal cord. At our facility, several patients presented to therapy planning with metastases involving or adjacent to the spinal cord with high ^131^I uptake. Such metastases merit special attention, with restrictions of the absorbed dose being determined according to the dimension of the irradiated area during radionuclide therapy procedures, such as external radiotherapy, such analysis having been the *modus operandi* for decades. It is also quite common to see patients presenting with lung metastases, a situation in which the lung, rather than the red marrow, is the limiting organ for determining the total amount of ^131^I to be applied in therapy. Therefore, the evaluation of tumor sites and dose restriction based on ^131^I biokinetics in a specific organ or tumor cannot be determined only by probe detection, in which the analysis and quantification of nuclear medicine images is the rule. Tailoring the amount of ^131^I to be administered to a patient based on biokinetics data obtained from probe detection alone could lead to the use of incorrect procedures, with the possibility of causing severe damage to the patient.

From our perspective, there is no doubt that the future of nuclear medicine dosimetry will involve image quantification, given the various advantages of its use in therapy planning, such as restoring the bases of radionuclide therapy, thus avoiding cell damage caused by the delivery of inappropriate radiation doses to a specific organ or tumor.

## CONCLUSIONS

There is strong positive agreement between the probe detection and image quantification methods for estimating ^131^I biokinetics and radiation doses to the red marrow and whole body for patients with metastatic thyroid cancer without metastatic bone involvement. Nevertheless, care should be taken when using the probe detection method, because it is unable to identify tumor sites and critical organs during therapy planning, which could have a negative impact on the adjustment of ^131^I amounts to be administered.
